# Bronchial Vascular Remodeling Is Attenuated by Anti-IL-17 in Asthmatic Responses Exacerbated by LPS

**DOI:** 10.3389/fphar.2020.01269

**Published:** 2020-09-04

**Authors:** Leandro do Nascimento Camargo, Tabata Maruyama dos Santos, Felipp Costa Pinto de Andrade, Silvia Fukuzaki, Fernanda Degobbi Tenorio Quirino dos Santos Lopes, Milton de Arruda Martins, Carla Máximo Prado, Edna Aparecida Leick, Renato Fraga Righetti, Iolanda de Fátima Lopes Calvo Tibério

**Affiliations:** ^1^Faculdade de Medicina FMUSP, Universidade de Sao Paulo, São Paulo, Brazil; ^2^Serviço de Reabilitação, Hospital Sírio-Libanês, São Paulo, Brazil; ^3^Department of Medicine, University City of São Paulo (UNICID), São Paulo, Brazil; ^4^Department of Bioscience, Federal University of São Paulo, Santos, Brazil

**Keywords:** anti-IL-17, vascular inflammation, LPS (lipopolysaccharide), asthma, vascular remodeling

## Abstract

**Introduction:**

Although the major alterations associated with asthma are related to the airways, there is also evidence of the importance of peribronchial vascular inflammation and remodeling in its pathophysiology.

**Objectives:**

To determine the effects of anti-IL-17 therapy on peribronchial vessels of an asthma model exacerbated by lipopolysaccharide.

**Methods:**

We evaluated several factors, including lung function, inflammation, oxidative stress, vascular remodeling, and signaling pathways present in the peribronchial vessels of 66 male BALB/c mice exposed to ovalbumin and treated (or not) treated with anti-IL-17. Twenty-four hours before the end of the experimental protocol, groups of sensitized animals (OVA–LPS and OVA–LPS anti-IL-17) also received LPS.

**Results:**

The OVA–LPS-anti-IL-17 group presented a decrease in several factors [airway resistance and elastance, bronchoalveolar lavage fluid (BALF) cell counts, inflammatory response, eosinophils, TSLP, IL-33, TARC, TNF-α, CD4+, CD8+, IL-4, IL-6, IL-10, IL-17, and VEGF positive cells/10^4^μm^2^, peribronchovascular edema, and angiogenesis], including remodeling (MMP-9, MMP-12, TIMP-1 and TGF-*β* positive cells and volume fraction of collagen fibers I, collagen fibers III, collagen fibers V, decorin, lumican, actin, biglycan, fibronectin, and integrin), oxidative stress (iNOS positive cells and volume fraction of PGF2*α*), and signaling pathways (FoxP3), as well as dendritic cells, NF-kB, ROCK-1, ROCK-2, STAT-1, and phosphor-STAT1-positive cells compared to OVA–LPS (p < 0.05).

**Conclusions:**

In this model of LPS-induced asthma exacerbation, IL-17 inhibition represents a promising therapeutic strategy, indicating the potential of bronchial vascular control of Th2 and Th17 responses and the activation of the remodeling and oxidative stress pathways, associated with the control of signaling pathways.

## Introduction

Pulmonary remodeling in asthma is considered to be the main cause of symptoms associated with decreased lung function, occurring due to an imbalance between the creation of lesions and tissue repair (Lazaar and Panettieri, 2003). Airway remodeling in asthma is known to be responsible for most of the morphofunctional changes. These changes are partially reversible in mild asthma but are often irreversible in severe asthma (Lazaar and Panettieri, 2003; Postma and Timens, 2006).

In recent years, vascular remodeling has aroused the interest of the scientific community as the main mechanism of the immune response to pathogens, organisms, and foreign antigens (Zanini et al., 2010). In addition, bronchial vessels also play important functions in maintaining tissue homeostasis, as well as the supply of oxygen and nutrients.

Studies have shown that changes in the vascular components result in airflow obstruction in chronic allergic inflammation due to the process of angiogenesis, which leads to an increase in the amount of new vessels, as suggested by vasodilation and cellular margination with transmigration to target tissues (Kotsimbos and Hamid, 1997; Hoshino et al., 2001). It is believed that these combined changes contribute to an increase in the thickness of the airway walls, thus reducing the lumen of the airway caused by bronchial hyperresponsiveness and leading to air trapping. These changes are associated with disease progression and have been found in both asthma and COPD (Walters et al., 2008).

Otani et al. (2004) demonstrated that during the process of chronic inflammation, new capillaries, which are immature and unstable, may contribute to increased permeability and therefore could be used as a potential marker for the severity of disease. The inflammatory process of the disease itself leads to vascular changes (Otani et al., 2004). This complex process initially involves the activation and gene expression, transcription, and translation at the molecular level of signaling pathways, which once activated release or potentiate an increase in neuropeptides, cytokines, inflammatory mediators of growth, eosinophilic granular proteins, and proteases. This inflammatory cascade leads to endothelial cell activation and a proliferative endothelial response (Postma and Timens, 2006; Zanini et al., 2010).

Clinical studies and experimental studies have revealed that structural changes in the components of the extracellular matrix (ECM) occur under chronic inflammatory conditions in both the airway and distal parenchyma (Araujo et al., 2008; Prado et al., 2011; Pigati et al., 2015). Araujo et al. (2008) demonstrated in biopsy of patients with severe asthma, increased levels of fibronectin and elastic fibers in the smooth muscle of the airway wall. And it is already known that the increase of the vessels and the number of these has the final consequence of the edema, attributed to a state of “leakage” of the bronchial microcirculation. This increased vasculature and this altered permeability state have implications for airway hyperreactivity.

During the remodeling process, increased microvascular permeability, edema, and angiogenesis occur (Evans et al., 1985). Although these characteristics were reported in asthma by the scientific community in the 1990, many studies have since demonstrated the importance of these changes in the search for therapeutic alternatives for the treatment of this disease (Postma and Timens, 2006).

The role of vascular endothelial growth factor (VEGF) was described in asthma for the first time by Hoshino et al. (2001). It is now known that VEGF is a potent angiogenic factor that is increased in asthma and is correlated with vascular permeability (Asai et al., 2002). However, several inflammatory mediators and members of the family of growth factors that induce angiogenesis have been identified (Puxeddu et al., 2005). Among them, VEGF is considered the most potent regulator of direct action angiogenesis, and, in both fibrosis and chronic allergic inflammation, there is an increased expression of this endothelial growth factor (Chetta et al., 2005).

What also explains the emergence of new vessels during chronic allergic inflammation is the lack of balance between angiogenic inhibitors and angiogenic inducers. An imbalance in favor of pro-angiogenic factors leads to the anomalous growth of new blood vessels, contributing to the engorgement of the vasculature and a thickening or stiffening of the airway wall, in which VEGF and inflammatory mediators are involved (Liekens et al., 2001; Postma and Timens, 2006).

It is worth mentioning that the Th2 pathway through innate lymphoid cell (ILC) activation and the IL-33-ST2 axis plays an important role in the development of asthma, contributing to the process of pulmonary remodeling in addition to having participation in the angiogenesis process, potentiating both vessel remodeling responses as the airway (Choi et al., 2009; Stojkovic et al., 2014; Shan et al., 2016; Puttur et al., 2019). Shan et al. (2016), evaluating the participation of IL-33-ST2, hypothesized that IL-33 is capable of inducing airway angiogenesis and expression of angiogenic factors (Shan et al., 2016).

In recent years, Th17 cells have gained the interest of the scientific community in relation to the modulation of chronic allergic inflammation since part of the population with non-allergic asthma tends not to respond to classical therapy (De Boever et al., 2014).

The Th 17 profile and cytokines they produce have played a key role in the development of neutrophilic asthma and are correlated with steroid resistant to disease severity (Al-Ramli et al., 2009; Camargo et al., 2018).

IL-17 (previously known as IL-17A) is an important pro-inflammatory cytokine of the Th17 pathway and was first identified in 1993. It is involved in neutrophilic inflammation and pulmonary remodeling processes (Al-Ramli et al., 2009).

Camargo et al. (2018) studied a model of allergic inflammation exacerbated by LPS in order to mimic the neutrophilic asthmatic profile and demonstrated the participation of Th17 cells in signaling pathways (inflammation, oxidative stress, and pulmonary remodeling). In the same study, the authors used the IL17A inhibitor to demonstrate that anti-IL-17 was able to control and attenuate the responses in these different pathways (Camargo et al., 2018).

Soon after, Dos Santos et al. (2018) associated two treatments in a model of chronic allergic inflammation to demonstrate the positive results both in the treatment alone and in the combination of anti-IL-17 and anti-Rho-kinase (Dos Santos et al., 2018). Both demonstrated benefits and could be used safely in animal models. However, the role of Th17 cells in vascular remodeling in asthma has not yet been elucidated.

The role of IL-17 in cardiovascular changes has been documented, although little is known. Studies on models of pulmonary hypertension show the contribution of this profile to changes in the pulmonary artery after hypoxemia (Chabot et al., 2009; Soon et al., 2010).

Despite few studies using anti-IL-17 in experimental models to evaluate its effect on pulmonary vascular remodeling, what has been documented are positive results both in the control of inflammation and in the control of vascular remodeling (Lu et al., 2015).

Sundrud and Trivigno (2013) reported the strong involvement of IL-17 with Jak-Stat family signaling, particularly STAT3, in many diseases (Sundrud and Trivigno, 2013). STAT3 is known to be involved in the remodeling process *via* IL-6, IL-8, and IL-17 stimulation. In addition, STAT3 signaling has been shown to be involved in VEGF production, wherein IL-17 directly activates the tyrosine phosphorylation of STAT1, STAT2, STAT3, and STAT4 in human monocytic leukemia cells (Subramaniam et al., 1999). However, there are currently no published works evaluating the participation of the STAT1/Th17 pathway in the vascular responses of chronic allergic inflammation.

However, to date, there are few studies evaluating the role of IL-17 in pulmonary vascular remodeling in models of allergic inflammation (Kang et al., 2012; Lu et al., 2015; Panariti et al., 2018).

Our aim was to evaluate the effect of anti-IL-17 on the different pathways inflammatory, oxidative stress, vascular remodeling in the peribronchial vessels in an experimental model of allergic inflammation exacerbated by LPS.

### Materials and Methods

This project was approved by the Research Ethics Committee of the Hospital das Clínicas of the Medical School of the University of São Paulo (protocol number 141/16). This work was developed in the Laboratory of Experimental Therapy I (LIM 20) of the Faculty of Medicine of the University of São Paulo.

### Experimental Groups

This protocol was repeated twice. Sixty six male BALB/c mice from the Faculty of Medicine of the University of São Paulo were utilized in accordance with the Guideline to Care and Use of Laboratory Animals published by the National Institutes of Health (NIH publication 85-23, revised in 1985). Mice aged 6 to 8 weeks with a mean weight of 20–28 g were divided into six groups:

1. SAL GROUP: mice received inhalations with a sterile saline solution (n = 6);
2. OVA GROUP: mice received IP and inhalations of an ovalbumin solution (n = 6);
3. OVA ANTI-IL-17 GROUP: mice received inhalations of an OVA solution and treatment with an anti-IL-17 monoclonal antibody (n = 6);
4. OVA–LPS GROUP: mice received inhalations of an ovalbumin solution and LPS instillation (n = 6);
5. OVA–LPS ANTI-IL-17 GROUP: mice received inhalations of an ovalbumin solution, LPS instillation, and treatment with an anti-IL-17 monoclonal antibody (n = 6).

We included a negative control (SAL anti-IL-17) when we repeated the protocol.

1. SAL-anti-IL-17—which received inhalations with a sterile saline solution and treatment with anti-IL-17 monoclonal antibody (n = 6).

### Ovalbumin Sensitization Protocol

**Figure 1:**
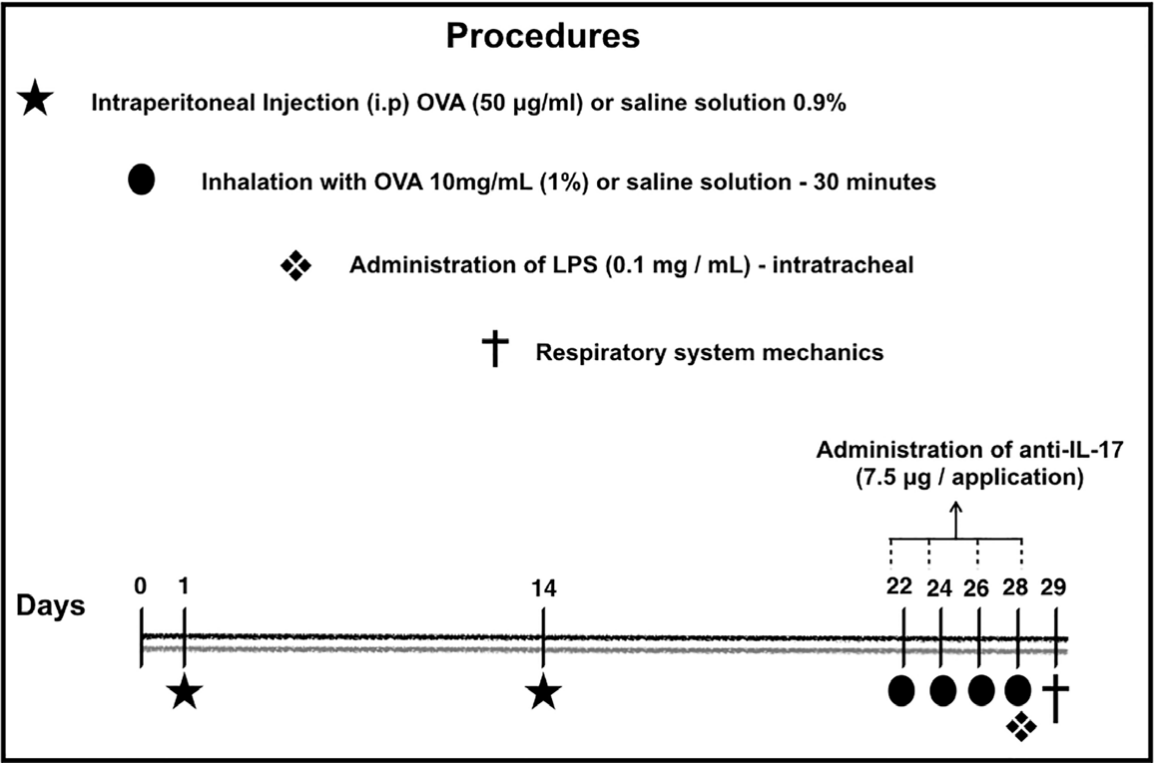
Timeline of the sensitization protocol. On days 1 and 14, the BALB/c mice received a solution of OVA intraperitoneally (i.p.). On days 22, 24, 26 and 28, the animals were submitted to inhalation for 30 min at 1% concentration. The control group received a saline solution i.p. and was exposed to 0.9% saline aerosol for 30 min as the inhalation challenge. Anti-IL-17 neutralizing antibody was administered i.p. 1 h prior to the intratracheal instillation of LPS. Twenty-four hours after the final antigen challenge, on day 29, the animals received LPS intratracheally. On the 29^th^ day, the mechanics of the respiratory system and bronchoalveolar lavage (Barlow et al., 2011; Starkhammar et al., 2012; Camargo et al., 2018).

The sensitization protocol lasted for 29 days. The protocol used in this study is shown in **Figure 1**. On days 1 and 14, the BALB/c mice received a solution of 50 µg of OVA (Sigma-Aldrich) and 6 mg of Al(OH)_3_ adjuvant (Pepsamar, Sanofi) intraperitoneally (i.p.) in a total volume of 0.2 ml (Synthelabo SA, Rio de Janeiro, Brazil). On days 22, 24, 26, and 28, the animals were submitted to inhalation for 30 min (ultrasonic nebulizer; US-1000, ICEL, São Paulo, Brazil) coupled to an acrylic box (30 × 15 × 20 cm) diluted in 0.9% NaCl at 1% concentration. At the same time, the control group and was administered a saline solution (NaCl 0.9%) i.p. and was exposed to 0.9% saline aerosol for 30 min for the inhalation challenge (Righetti et al., 2014; Pigati et al., 2015).

### LPS Sensitization

The administration of LPS was carried out according to the protocol provided by Starkhammar et al. (2012) and Camargo et al. (2018). The treatment was performed with 20 μl of PBS + 0.1 mg/ml *Escherichia coli* 0127:B8 (Sigma-Aldrich, St Louis, MO, USA) 24 h after the last antigen challenge on day 29. The animals in the LPS–OVA and anti-LPS OVA IL-17 groups were anesthetized *via* the inhalation of isoflurane and were administered LPS intratracheally. No animal died during the protocol.

### Anti-IL-17 Treatment

Anti-IL-17 neutralizing antibody (R&D Systems, Abingdon, UK) was administered i.p. at a dose of 7.5 μg/application, based on the protocol provided by Barlow et al. (2011) and Camargo et al. (2018) 1 h prior to the intratracheal instillation of LPS.

### Exhaled Nitric Oxide (NOex) and Respiratory System Mechanics

On the 29th day, animals were anesthetized with thiopental (50 mg/kg i.p.) and tracheostomized. After tracheostomy, we attached the Harvard 683 ventilator (Harvard Apparatus, South Natick, MA, United States) and adjusted the following parameters: tidal volume of 10 ml/kg, respiratory rate of 120 cycles/min, and sinusoidal inspiratory flow curve. To abolish the ventilatory effort, the animals received pancuronium (0.2 mg/kg i.p.) (Righetti et al., 2014). For NOex collection, the gas was collected in the ventilator’s expiratory portion through a nitric oxide-impermeable balloon (Mylar Bag, Sievers, Instruments Inc., Boulder, CO, USA), for 10 min as standardized in our laboratory. A sample was collected immediately after the animals were connected to the mechanical ventilator and stabilized. After the end of the collection period, the balloons were sealed for further analysis. Nitric oxide was measured by chemiluminescence using a rapid response analyzer (280 NOA—Nitric Oxide Analyzer—Sievers Instruments Inc., Boulder, CO). The average concentration of nitric oxide was recorded in parts per billion (ppb) as an index of the concentration of nitric oxide in the exhaled air. After collecting the exhaled nitric oxide balloon, the animals were subjected to an evaluation of the respiratory system mechanics (Bittencourt-Mernak et al., 2017).

The assessment of methacholine hyperresponsiveness was initiated using the LABDAT software (RHT-InfoData, Montreal, Quebec, Canada). To measure tracheal pressure (Ptr), the 142PC05D differential pressure transducer (Honeywell, Freeport, IL) was used, while flow (V′) was measured by a pneumotachograph (Fleisch-4.0, OEM Medical, Richmond, VA, USA); both were connected to the tracheal cannula. For an accurate definition of changes in lung volume (V), an electronic system was used to integrate the flow circuit. We use computers to store and collect traces of Ptr, V′ and V. These values were used to calculate the basal and maximum resistance (Rrs) and elastance (Ers) of the respiratory system after the administration of methacholine aerosol (3, 30, and 300 mg/ml, for 1 min). The resistance and elastance values of the respiratory system were calculated through the equation of respiratory system movement, described below: Ptr (time) = Rrs.V′ (time) + Ers.V (t), where: Ptr is tracheal pressure, Rrs is resistance, Ers is elastance, V′ is airflow, V is lung volume, and t is time. After the evaluation of respiratory mechanics, the animals were euthanized by exsanguination of the abdominal aorta (Righetti et al., 2014; Dos Santos et al., 2018).

### Bronchoalveolar Lavage

After the respiratory mechanics were evaluated, bronchoalveolar lavage was performed. Saline solution (0.5 ml each) was instilled three times with a syringe through the tracheostomy cannula and a total volume of 1.5 ml was recovered. The BALF was centrifuged at 790× g for 10 min at 5°C with an average mean recovery of 80% (Camargo et al., 2018).

The cell pellet was resuspended in 300 μl of saline using a vortex. Then, 100 μl was used to prepare a slide for differential cell counting. The remaining BALF was centrifuged onto a slide in the cytospin for 6 min at 450 rpm and then stained with diff quick. Total cell counts were performed by light microscopy with the Neubauer hemocytometer (400×). Differential cell count of neutrophils was performed using an optical light microscope at 1,000× magnification (Camargo et al., 2018).

### Histological Analysis

The hearts and lungs were removed and stored. Lungs were fixed with 4% formaldehyde at a constant pressure of 20 cmH_2_O for 24 h, then maintained in 70% alcohol for up to 36 h and finally prepared for histological processing. For this, pulmonary tissue fragments were fixed and embedded in paraffin. Five-micrometer thick slices were fixed on sheets and prepared with 3-aminopropyl-triethoxysilane Silane (Sigma) containing the histological sections of the lungs. The slices were deparaffinized, rehydrated, and treated with Proteinase K for 20 min (37°C) followed by standing for 20 min at room temperature before washing with phosphate-buffered saline (PBS). Blocking endogenous peroxidase was performed by incubation with 3% hydrogen peroxide (H_2_O_2_) 10 V (3 × 10 min).

**Figure 2:**
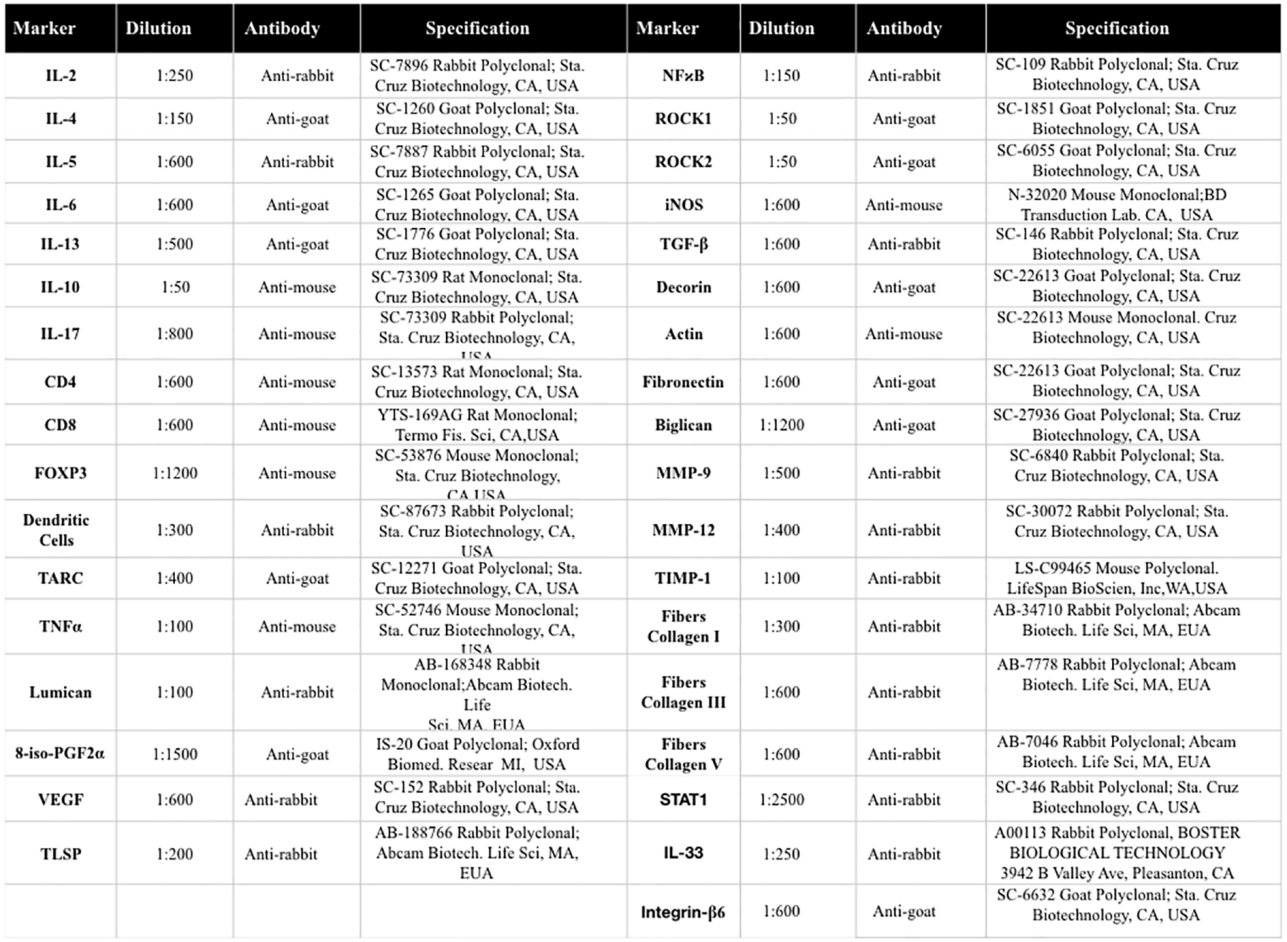
Description of antibodies used for immunohistochemistry analysis.

The slices were prepared in silanized leaves for subsequent immunohistochemistry to evaluate the vascular areas. For incubation with the primary antibody, the primary antibodies were diluted in bovine serum albumin (BSA) solution and applied to each slice. The antibodies used and their dilutions and tags are provided in **Figure 2**. The slides were incubated overnight in a humid chamber in a refrigerator at 4°C.

After 18 to 22 h, the slides were washed in PBS and incubated with the secondary antibody. The slides were washed in PBS, and proteins were visualized using diaminobenzidine chromogen (DAB) (Sigma Chemical Co., St. Louis, MO, USA). The slide sections were counterstained with Harris hematoxylin (Merck, Darmstadt, Germany) and mounted with microscopy resin. For the evaluation of eosinophils, the lungs were stained with Luna (Direct Red 80, C.I.35780, Aldrich, Milwaukee, WI, USA) stain for eosinophil granules (Watanabe et al., 2004).

### Morphometric Analysis

For the morphometric evaluation, we evaluated the cell density, TSLP, IL-33, TARC, TNF-*α*, CD4+, CD8+, IL-4, IL-6, IL-10, IL-17, VEGF, MMP-9, MMP-12, TIMP-1, TGF-*β*, iNOS, FoxP3, dendritic cells, NF-kB, ROCK-1, ROCK-2, STAT-1, and phospho-STAT1 in the bronchial vessels. These techniques were performed by two different researchers blinded to the protocol design, as described below. The dot counting technique was employed by means of a grid of a known area (50 lines and 100 points) coupled to a microscope (E200MV; Nikon Corporation, Tokyo, Japan) (Dos Santos et al., 2018). Three random fields were evaluated around the vessel with a total of five bronchial vessels per animal counted at a magnification of 1,000×. The number of positive cells was established based on the number of points that coincided with the positive cells within the reticulum divided by the number of points coincident with the lung tissue. The total area of the reticulum was 10^4^ μm^2^. The analysis was performed at a magnification of 1,000× (Angeli et al., 2008; Nakashima et al., 2008).

Optical density was used to evaluate the volumetric vascular fractions of type I, III, and V collagen fibers, actin, decorin, lumicam, fibronectin actin, biglycan, integrin, and isoprostane PGF2*α*. The images were captured using a microscope (Leica DM2500; Leica Microsystems, Wetzlar, Germany) mounted with a digital camera (Leica DFC420; Leica Microsystems, Wetzlar, Germany). The images were acquired and processed using Optimas v.4.10 software. We analyzed three fields in a total of five vessels per animal. The images were analyzed using Image-Proplus 4.5 software (NIH, Maryland, USA). This software allowed for a threshold of the color tones to be developed. The shades represented the positive quantified areas in the predetermined area. The volumetric vascular fractions of these markers are expressed as percentages of the area (Righetti et al., 2014).

To evaluate the angiogenesis, we used the point counting technique. The number of vessels and the number of points that were coincident with lung tissue were counted, and the results were expressed as the number of vessels per area (10^4^ μm^2^) (Prado et al., 2011).

The evaluation of peribronchial edema was performed using hematoxylin and eosin (H&E) staining. Quantifications were performed according to the point counting technique described above. The results were expressed in terms of the size of the area of the edema (μm^2^) (Pinheiro et al., 2015).

### Statistical Analysis

All data are presented as the mean ± standard error (SE). Graphs are presented in bar format. One-way analysis of variance (ANOVA) followed by the Holm–Sidak method for multiple comparisons was used to determine the difference between groups with statistical significance. All analyses were conducted using SigmaPlot 11.0 software (Systat Software, SPSS Inc., USA). A *p*-value < 0.05 was considered statistically significant.

## Results

The protocol was repeated and we included the anti-IL-17 SAL group. We performed the comparison with the SAL group on the following markers, IL-4 (0.58 ± 0.24) *vs* (1.17 ± 0.14), IL-13 (0.43 ± 0.11) *vs* (0.12 ± 0.03), IL-17 (1.68 ± 0.52) *vs* (0.39 ± 0.15) and iNOS (1.01 ± 0.19 0.52 ± 0.07); we did not observe differences between the groups so we decided to only evaluate the SAL group in the other markers, except neutrophils and pulmonary mechanics.

### Effect of Anti-IL-17 Treatment on Vascular Remodeling

**Figure 3:**
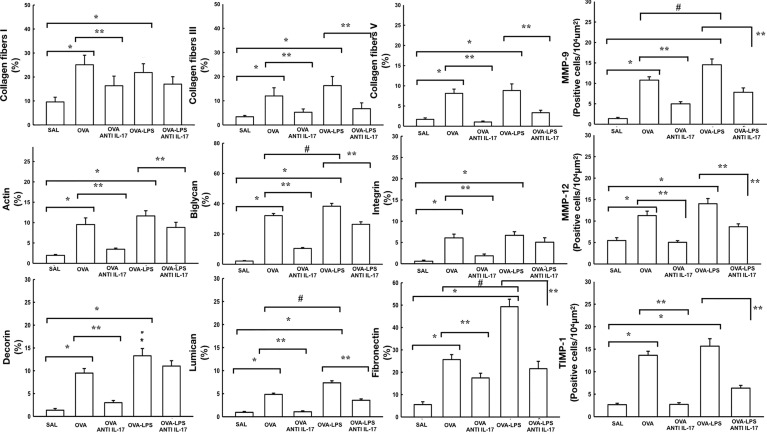
Effects of anti-IL 17 treatment on vascular remodeling. The values represent the mean ± standard error (SE) of the collagen fibers I, III, V, MMP-9, actin, biglycan, integrin, MMP-12, decorin, lumican, fibronectin, and TIMP-1 for all experimental groups. The differences were considered significant when p < 0.05. *p < 0.05 *vs.* SAL group; **p < 0.05 *vs.* OVA and OVA–LPS groups. ^#^p < 0.05 *vs.* OVA group.

**Figure 4:**
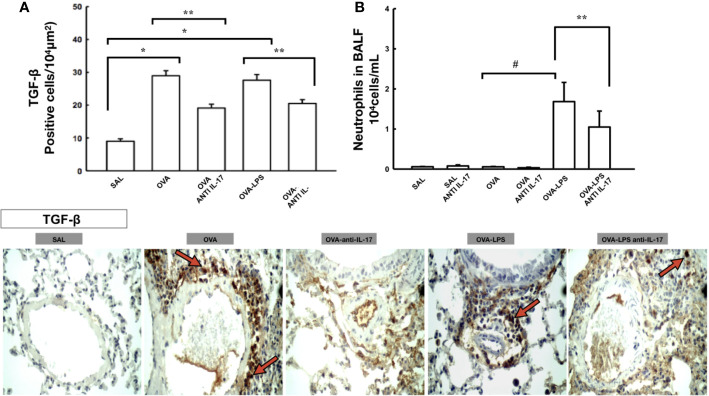
Effects of anti-IL 17 treatment on TGF-*β* and neutrophils in BALF. Panel **A** shows the expression of positive cells for TGF-β in the pulmonary vessels and Panel **B** shows the number of neutrophil cells in the bronchoalveolar lavage fluid. The values represent the mean ± SE of TGF-*β* and neutrophils for all experimental groups. The differences were considered significant when p < 0.05. *p < 0.05 *vs.* SAL group; **p < 0.05 *vs.* OVA and OVA–LPS groups. ^#^p < 0.05 *vs.* OVA group. Photomicrograph pulmonary vessels measuring TGF-*β*. The animals exposed to only ovalbumin and ovalbumin plus LPS (OVA and OVA–LPS groups) presented prominent increases in the numbers of positive cells, compared with the control animals (SAL group). Anti-IL-17 treatment in the OVA anti-IL-17 and OVA–LPS anti-IL-17 groups ameliorated all increases in these markers. The red arrows indicate the positive cells for TGF-*β*.

**Figure 3** shows the effects of anti-IL-17 treatment on the remodeling of the pulmonary vessel wall contents for collagen fibers I, III, V, biglycan, decorin, actin, lumican, integrin, fibronectin and number of cells positive for MMP-9, MMP-12, and TIMP-1 expression. For expression of TGF-*β* positive cells in the pulmonary vessels are shown in **Figure 4A**. We found that the OVA and OVA–LPS groups showed an increase in MMP-9, MMP-12, TIMP-1 positive cells, as well as an increase in the collagen fiber I, III, and V fractions, Biglycan, decorin, lumican, fibronectin, actin, and integrin, and actin compared to the SAL group (p < 0.05). The OVA–LPS group showed an increase compared to the OVA group (p < 0.05), except for MMP-12, TIMP-1, collagen fibers I and III, integrin, lumican, and actin. The animals treated with anti-IL-17 showed a reduction in all of the markers (OVA anti-IL-17 and OVA–LPS anti-IL-17 groups) compared to the OVA and OVA–LPS groups respectively (p < 0.05). Only collagen fiber I, lumican, decorin, and integrin did not present any reduction in OVA–LPS anti-IL-17 compared to the OVA–LPS group.

### Effects of Anti-IL 17 Treatment on TGF-β and Neutrophils in BALF

For expression of TGF-β positive cells in the pulmonary vessels are shown in **Figure 4A** and neutrophils bronchoalveolar lavage is shown in **Figure 4B**. There was an increase in the number of TGF-β positive cells in the pulmonary vessel wall and in the number of neutrophils in the bronchoalveolar lavage in the OVA and OVA-LPS groups compared to the SAL group (p < 0.05).

### Effect of Anti-IL-17 Treatment on Rrs, Ers, Angiogenesis, Peribronchovascular Edema, and VEGF

**Figure 5:**
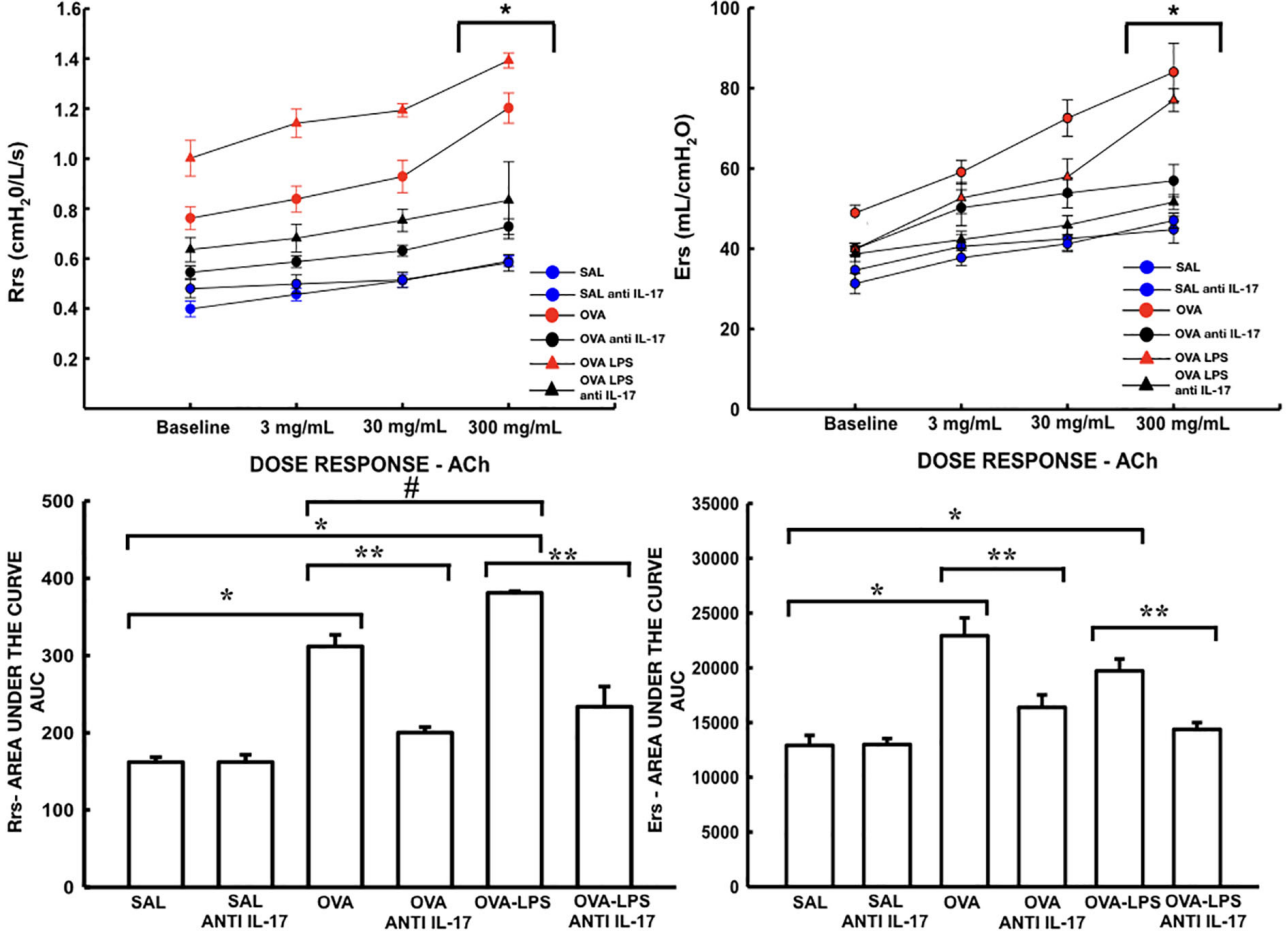
Effects of anti-IL-17 treatment on resistance (Rrs) and elastance (Ers). Dose/response curves of methacholine dose/3, 30, and 300 mg/ml. Data are presented as mean ± sem. The differences were considered significant when p < 0.05. *p < 0.05 *vs.* baseline. Values of the logarithm of the area under the curve (AUC) of resistance (Rrs) and elastance of the respiratory system (Ers) were considered a significant difference when p < 0.05. *p < 0.05 *vs.* SAL group; **p < 0.05 *vs.* OVA and OVA–LPS groups. #p < 0.05 *vs.* OVA group.

**Figure 6:**
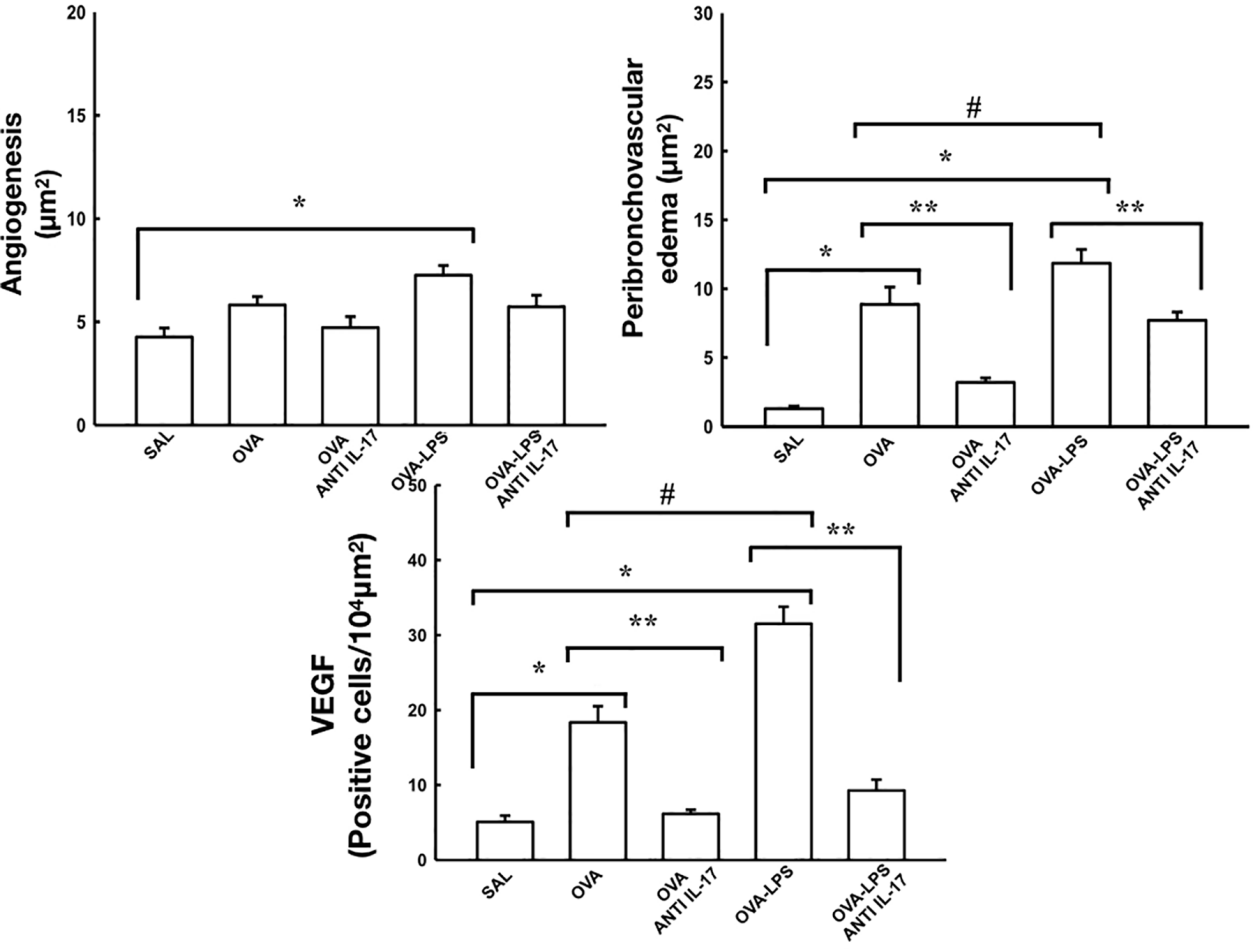
Effects of anti-IL-17 treatment on angiogenesis, peribronchovascular edema, and vascular endothelial growth factor (VEGF). The values represent the mean ± standard error (SE) of the angiogenesis, peribronchovascular edema, and vascular endothelial growth factor (VEGF) for all experimental groups. The differences were considered significant when p < 0.05. *p < 0.05 *vs.* SAL group; **p < 0.05 *vs.* OVA and OVA–LPS groups. ^#^p < 0.05 *vs.* OVA group.

The resistance and elastance are presented in **Figure 5**. The quantification of new vessels, peribronchovascular edema, and positive cells for VEFG is shown in **Figure 6**. There was an increase in the peribronchovascular edema and number of positive cells for VEGF expression in the OVA and OVA–LPS groups compared to the SAL control group (p < 0.05). There was an increase in the number of new vessels only in the OVA–LPS group compared to the SAL control group (p < 0.05). The OVA–LPS group showed an increase compared to the OVA group for the same parameters (p < 0.05), except for the number of new vessels. The animals treated with anti-IL-17 showed a reduction in peribronchovascular edema and VEGF in the groups (OVA anti-IL-17 and OVA–LPS anti-IL-17) compared to the OVA and OVA–LPS groups, respectively (p < 0.05). There was no difference in the treatment groups for angiogenesis. When evaluating the dose/response curve, we observed that there was an increase in resistance and elastance of the respiratory system at baseline when compared to the maximum dose for all groups, except the anti-IL-17 OVA–LPS group. When we evaluated the area under the dose/response curve, we observed an increase in resistance and elastance of respiratory system in the OVA and OVA–LPS groups compared to the SAL control group (p < 0.05). The OVA–LPS group showed an increase only for resistance compared to the OVA group (p < 0.05). The treatment with anti-IL-17 attenuated the resistance and elastance of the respiratory system in the groups (OVA anti-IL-17 and OVA–LPS anti-IL-17) compared to the OVA and OVA–LPS groups, respectively (p < 0.05).

### Effect of Anti-IL-17 Treatment on Vascular Inflammation

**Figure 7:**
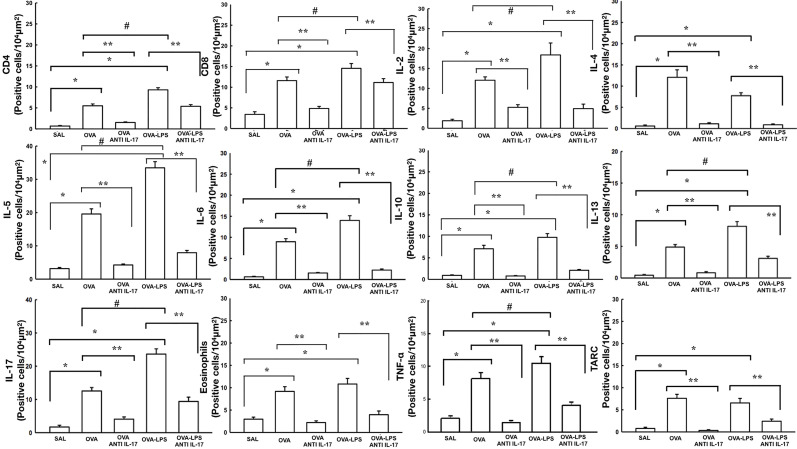
Effects of anti-IL 17 treatment on vascular inflammation. The values represent the mean ± SE of CD4, CD8, IL-2, IL-4, IL-5, IL-6, IL-10, IL-13, IL-17, eosinophils, TNF-α, and TARC for all experimental groups. The differences were considered significant when p < 0.05. *p < 0.05 *vs.* SAL group; **p < 0.05 *vs.* OVA and OVA–LPS groups. ^#^p < 0.05 *vs.* OVA group.

**Figure 8:**
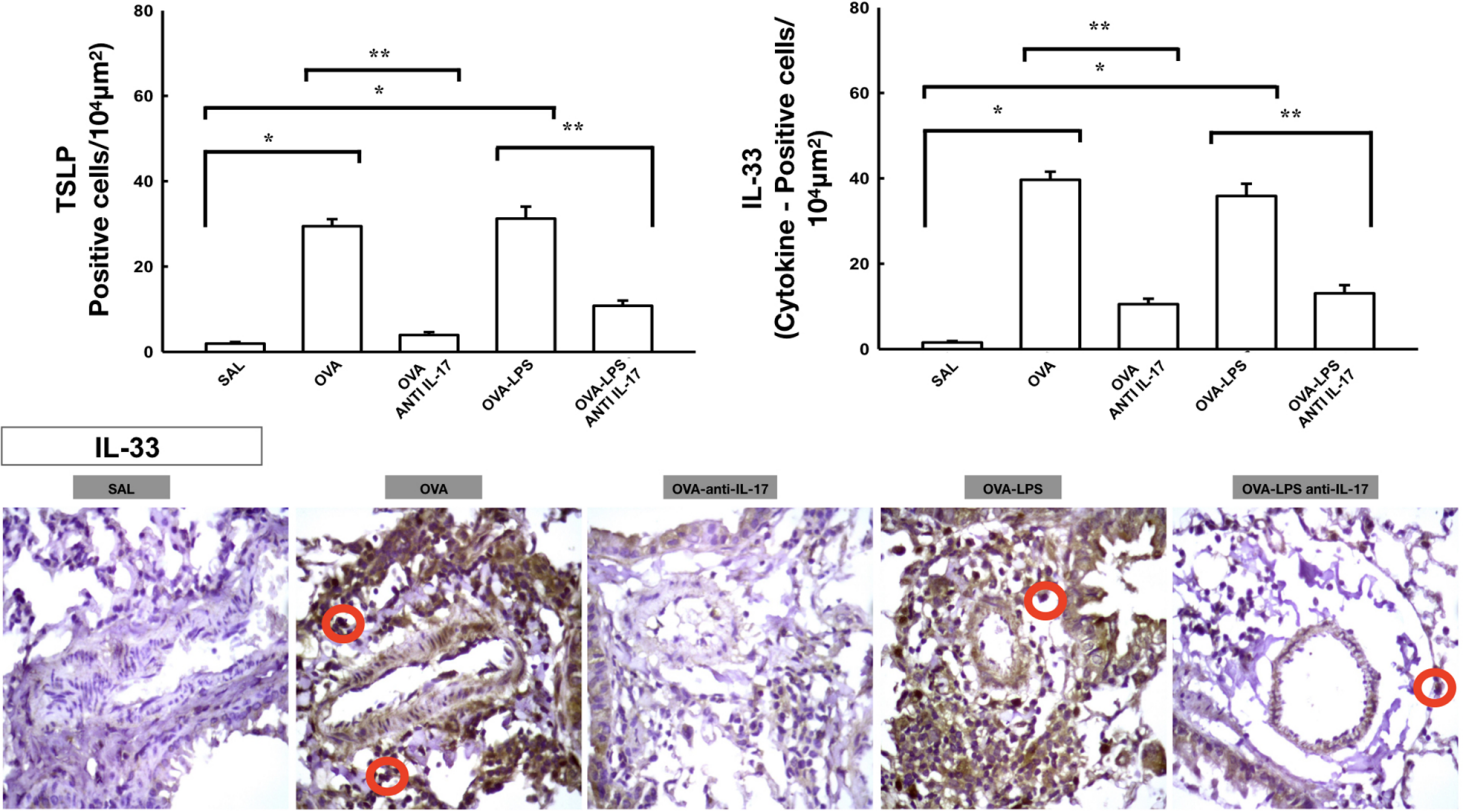
Effects of anti-IL 17 treatment on TSLP and vascular IL-33. The values represent the mean ± SE of TSLP and IL-33 for all experimental groups. The differences were considered significant when p < 0.05. *p < 0.05 *vs.* SAL group; **p < 0.05 *vs.* OVA and OVA–LPS groups. Photomicrograph pulmonary vessels measuring IL-33. The animals exposed to only ovalbumin and ovalbumin plus LPS (OVA and OVA–LPS groups) presented prominent increases in the numbers of positive cells, compared with the control animals (SAL group). Anti-IL-17 treatment in the OVA anti-IL-17 and OVA–LPS anti-IL-17 groups attenuated all increases in these markers. The IL-33 positive cells are identified by the red circle.

The number of differential cells in the bronchoalveolar lavage for neutrophils is shown in **Figure 4B**. The number of cells positive for CD4, CD8, IL-2, IL-4, IL-5, IL-6, IL-10, IL-13, IL-17, IL-33, TLSP, eosinophils, and TARC in the pulmonary vessel wall is presented in **Figures 7** and **8**. The number of positive cells for these markers in the pulmonary vessel wall was higher in the OVA and OVA–LPS groups compared to the SAL group (p < 0.05) for all comparisons. The OVA–LPS group showed a greater increase in these positive cells compared to the OVA group (p < 0.05), except for IL-4, IL-33, TSLP, and TARC. The number of positive cells for these markers was lower in the treated groups (OVA anti-IL-17 and OVA–LPS anti-IL-17) than in the OVA and OVA–LPS groups, respectively (p < 0.05). The expression of CD8 positive cells in the OVA–LPS anti-IL-17 group was not increased compared to OVA–LPS group.

### Effect of Anti-IL-17 Treatment on Antigen Presenting Cells (APC) and FoxP3+

**Figure 9:**
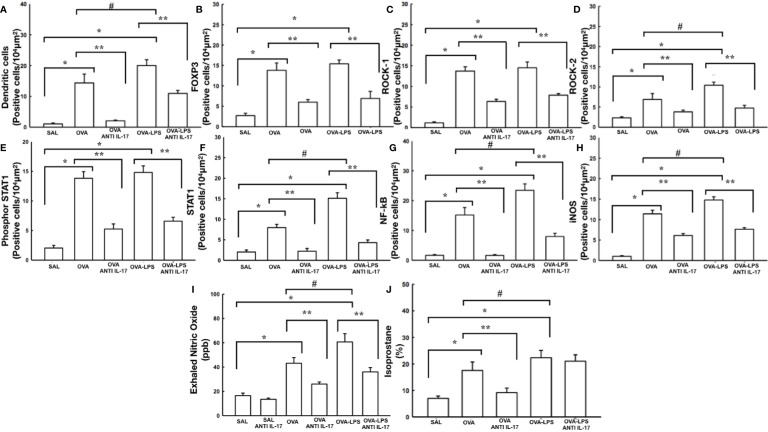
Effects of anti-IL 17 treatment on antigen-presenting cells **(A)**, FOxP3 **(B)**, signaling pathways **(C–G)**, and oxidative stress **(H–J)**. The values represent the mean ± SE of dendritic cells, FoxP3, ROCK-1, ROCK-2, Phosphor-STAT1, STAT1, NF-k, iNOS, NOex, and isoprostane for all experimental groups. The differences were considered significant when p < 0.05. *p < 0.05 *vs.* SAL group; **p < 0.05 *vs.* OVA and OVA–LPS groups. ^#^p < 0.05 *vs.* OVA group.

The cells positive for dendritic cells and FoxP3 in the pulmonary vessels in all experimental groups are presented in **Figures 9A, B**. There was an increase in the number of these positive cells in the pulmonary vessel wall in the OVA and OVA–LPS groups compared to the SAL group (p < 0.05). The OVA–LPS group was found to overexpress positive cells compared to the OVA group (p < 0.05). The number of positive cells for these markers was lower in the treated groups (OVA anti-IL-17 and OVA–LPS anti-IL-17) compared to the OVA and OVA–LPS groups, respectively (p < 0.05).

### Effect of Anti-IL-17 Treatment on Signaling Pathways

The number of ROCK-1, ROCK-2, phosphor-STAT-1, STAT-1, and NF-kB positive cells in the pulmonary vessels is shown in **Figures 9C–G**. There was an increase in the levels of ROCK-1, ROCK-2, NF-kB, STAT-1, and phospho-STAT1 expression in the pulmonary vessels in the OVA and OVA–LPS groups compared to the SAL group (p < 0.05). The OVA–LPS group presented a greater cell number potentiation than the OVA group (p < 0.05). No differences were found in terms of the number of ROCK-1-positive cells between the OVA–LPS and OVA groups. The anti-IL-17-treated groups showed an attenuation in the number of cells positive in the OVA anti-IL-17 and OVA LPS anti-IL-17 compared to the OVA and OVA–LPS groups, respectively (p < 0.05).

### Effect of Anti-IL-17 Treatment on Vascular Oxidative Stress

The increase in the volume fraction of isoprostane, iNOS positive cells in the pulmonary vessel and NOex can be observed in **Figures 9H–J**. These alterations were observed in the OVA and OVA–LPS groups compared to the SAL group (p < 0.05). The OVA–LPS group presented increased levels compared to the OVA group (p < 0.05). The anti-IL-17-treated groups showed an increase in these parameters in the OVA anti-IL-17 and OVA–LPS anti-IL-17 groups compared to the OVA and OVA–LPS groups, respectively (p < 0.05). The volume fraction of isoprostane in the OVA–LPS anti-IL-17 group was not reduced compared to the OVA–LPS group. There was no difference between the SAL and SAL anti-IL-17 groups.

### Qualitative Analysis

**Figure 10:**
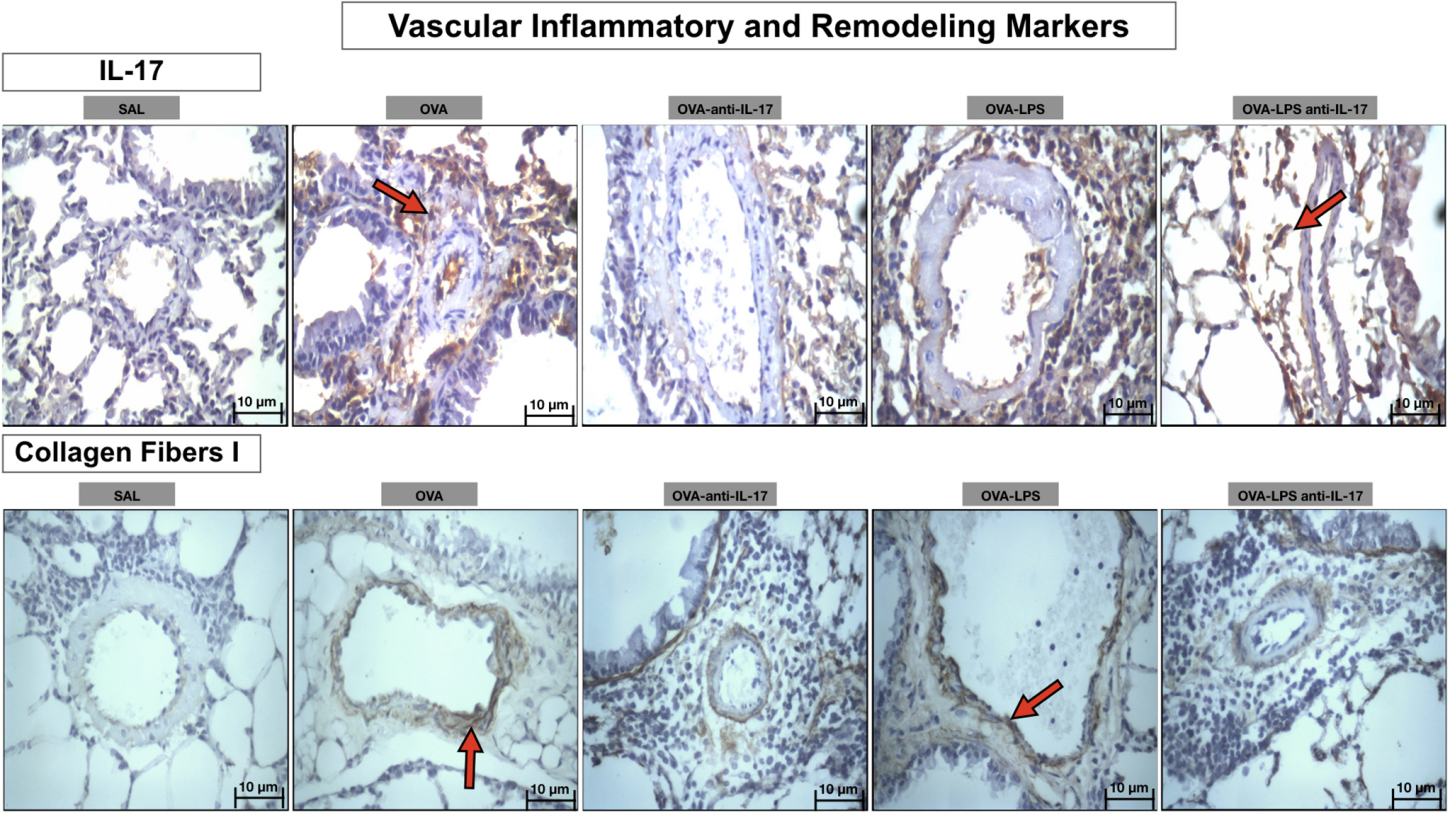
Qualitative analysis of vascular inflammation and remodeling. Photomicrographs showing the presence of inflammation around the pulmonary vessels. The slides were stained for IL-17 expressing positive cells and collagen fiber I content. The experimental groups include SAL, OVA, OVA-anti-IL-17, OVA–LPS, and OVA–LPS-anti-IL-17. The red arrows indicate the positive cells for IL-17 and collagen fiber I content.

Photomicrographs showing the presence of inflammation and remodeling of the pulmonary vessel wall expressed by IL-17 and collagen fibers- type 1 are shown in **Figure 10**. Animals exposed only to ovalbumin and ovalbumin plus LPS (OVA and OVA-LPS groups) showed prominent increases in the number of positive cells compared to control animals (SAL group). Treatment with anti-IL-17 in the OVA anti-IL-17 and OVA-LPS anti-IL-17 group attenuated these markers in both groups, except for collagen fibers- type 1 in the OVA-LPS anti-IL-17 group.

**Figure 11:**
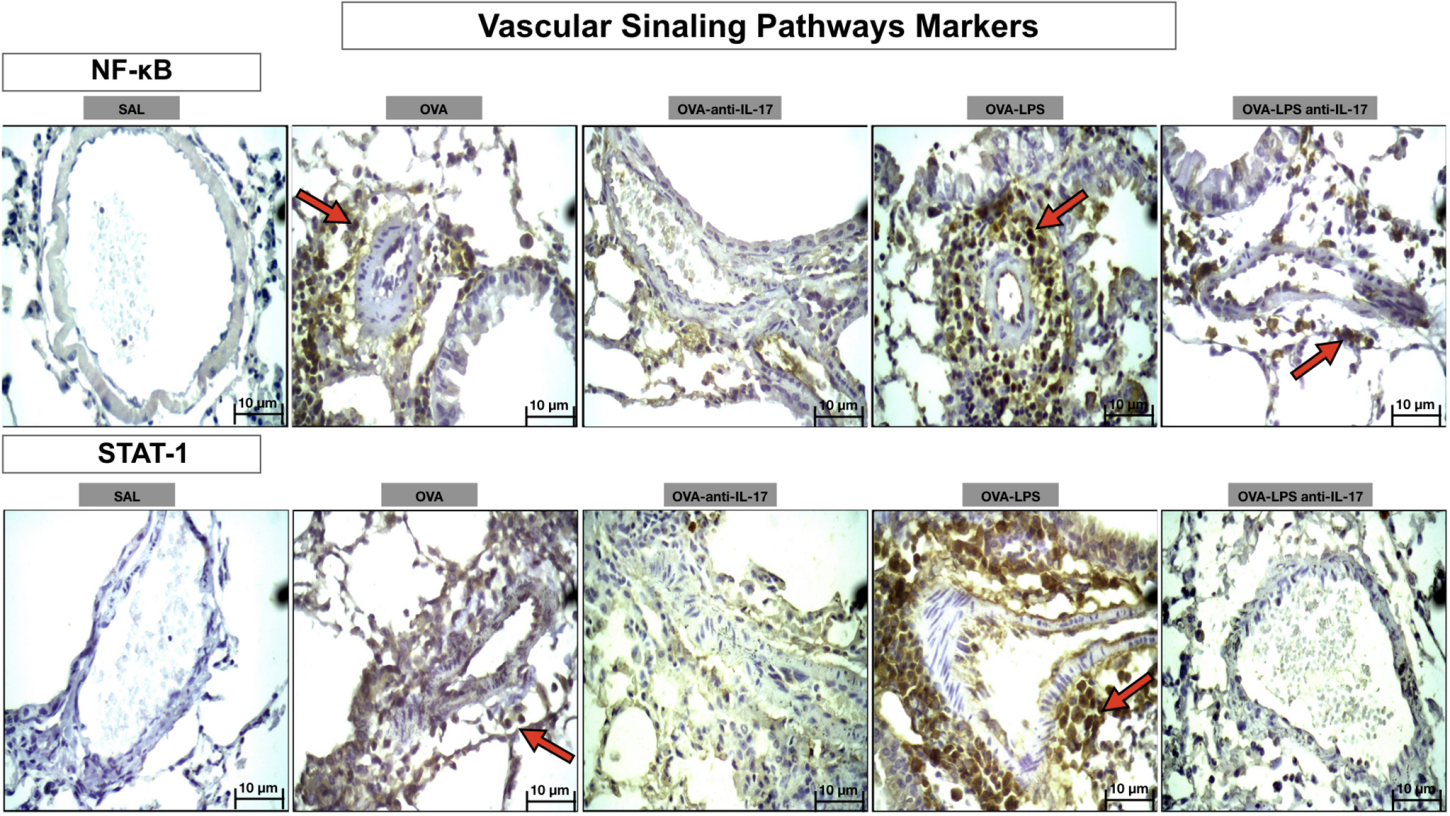
Qualitative analysis of vascular signaling pathways. Photomicrographs showing the presence of inflammation around the pulmonary vessels. The slides were stained for NF-kB and STAT-1-expressing positive cells. The experimental groups include SAL, OVA, OVA-anti-IL-17, OVA–LPS, and OVA–LPS-anti-IL-17. The red arrows indicate the positive cells for NF-kB and STAT-1.

Photomicrographs showing the presence of signaling pathway markers around the pulmonary vessels in **Figure 11**. Animals exposed only to ovalbumin and ovalbumin plus LPS (OVA and OVA-LPS groups) showed prominent increases in the number of positive cells compared to control animals (SAL group). Treatment with anti-IL-17 in the OVA anti-IL-17 and OVA-LPS anti-IL-17 group attenuated these markers in both groups.

**Figure 12:**
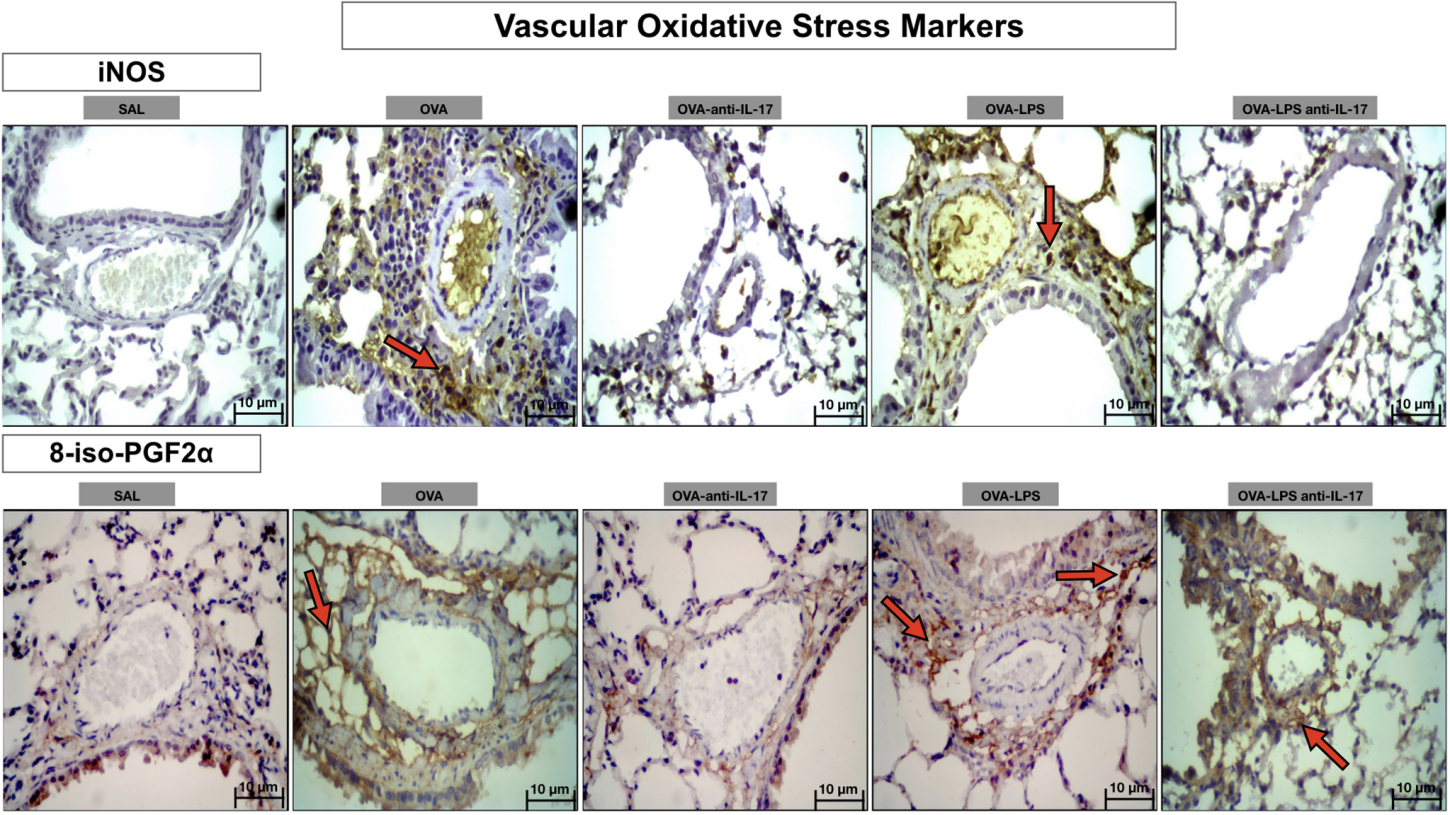
Qualitative analysis of vascular stress oxidative. Photomicrographs showing the presence of inflammation around the pulmonary vessels. The slides were stained for iNOS expressing positive cells and isoprostane content. The experimental groups include SAL, OVA, OVA-anti-IL-17, OVA–LPS, and OVA–LPS-anti-IL-17. The red arrows indicate the positive cells for iNOS and isoprostane content.

Photomicrographs showing the presence of stress oxidative markers around the pulmonary vessels in **Figure 12**. Animals exposed only to ovalbumin and ovalbumin plus LPS (OVA and OVA-LPS groups) showed prominent increases in the number of positive cells compared to control animals (SAL group). Treatment with anti-IL-17 in the OVA anti-IL-17 and OVA-LPS anti-IL-17 group attenuated these markers in both groups, except for 8-iso-PGF2α in the OVA-LPS anti-IL-17 group.

## Discussion

### Effect of Anti-IL-17 Treatment on Vascular Remodeling

In the present study, we assessed the role of changes in vascular remodeling in allergic inflammation exacerbated by LPS, as well as the role of anti-IL-17, an inhibitor of IL-17, in vascular remodeling. The results showed that the inhibition of IL-17 contributed to the control and attenuation of the content of collagen fibers I, III and V, lumicam, decorin, actin, biglican, fibronectin, integrin and TGF-*β*, TIMP-1, MMP-9, and MMP-12 positive cells.

Considered as the main marker for remodeling, collagen is synthesized *via* the stimulation of the transforming growth factor beta (TGF-*ß*). In cases of severe asthma, an increase in the deposition of collagens I, III, and V has been reported in bronchial biopsies (Chakir et al., 2003). In addition to participating in the inflammatory process, TGF-*β* is involved in changes to the extracellular matrix, stimulating the production of fibroblasts, collagen fibers, fibronectin, and proteoglycans, and inducing VEGF *via* endothelial cells, resulting in alterations to the vascular architecture (Puxeddu et al., 2005; Camargo et al., 2018). Studies evaluating aspects of airway remodeling and pulmonary parenchyma are in line with the increase in the markers assessed in this study (Kang et al., 2012; Hayashi et al., 2013; Shan et al., 2016; Camargo et al., 2018; Dos Santos et al., 2018). Despite few studies evaluating the characteristics of vascular remodeling in asthma, this study contemplates several remodeling markers showing the importance of vascular changes in allergic inflammation. It is worth mentioning that treatment with anti-IL-17 was able to attenuate all markers in the allergic inflammation model, and in the exacerbation model; only type I, lumican, decorin, and integrin collagen fibers were not attenuated. Future studies with protocol extension may show positive effects on these markers.

### Effect of Anti-IL-17 Treatment on Lung Function, Angiogenesis, Peribronchovascular Edema, and VEGF

In the aspect of Rrs and Ers, angiogenesis, peribroncoalveolar edema and VEGF, there was an increase in the OVA and OVA–LPS group, while treatment with anti-IL-17 was effective in controlling and attenuating these markers, except for angiogenesis, where there was no difference between the treatment groups compared to the OVA and OVA–LPS.

The first observations reported in the literature on vascular changes in severe asthma were described by Dunnill in 1960. Soon after, Dunnill et al. (Vieira et al., 2008) found that patients dying of severe asthma had edema in the bronchial mucosa with dilated and congested blood vessels (Vieira et al., 2008). Over time, our understanding of the modifications of this compartment and its contribution to the functional morphofunctional in asthma has only increased. Furthermore, in recent years, this had captured the interest of the scientific community since it is believed that these alterations amplify the functional effects of asthma, in addition to contributing to modifications of the pre-existing airway wall architecture (Wang and Wang, 2003; Wilson and Kotsimbos, 2003).

Plasma extravasation occurs during the inflammatory process, compromising epithelial integrity (Goldie et al., 1995). Studies have shown that plasma leakage during the process of chronic allergic inflammation contributes to the development of mucosal edema and a thickening of the bronchial wall thus reducing the lumen of the airways, which, in turn, leads to a reduction in airflow, which may contribute to airway hyperresponsiveness (Vieira et al., 2008).

Due to these changes already documented in the literature, we decided to evaluate the functional characteristics of respiratory mechanics, assessing airway hyperresponsiveness. Considering the index of the position of the dose/response curve (sensitivity) and the variation in the maximum response (reactivity), we observed an increase in the maximum response in comparison with the baseline for Rrs and Ers, and the treatment with anti-IL-17 managed to mitigate those responses. Our group previously demonstrated that experimental models show bronchial hyperresponsiveness when sensitized with ovalbumin and challenged with methacholine (Zanini et al., 2010; Righetti et al., 2014; Chan et al., 2019). Dos Santos et al. (2018) demonstrated that treatment with anti-IL-17 was able to attenuate hyperresponsiveness in animal models (Dos Santos et al., 2018). In addition, we used the LPS sensitization protocol provided by Starkhammar et al. (2012), which was demonstrated to increase hyperresponsiveness with LPS treatment (Starkhammar et al., 2012).

Panariti et al. (2018) demonstrated that an increase in bronchial vascularization and asthma severity correlates positively with the number of IL-17A+ cells, and the effects of IL-17A on vascular remodeling in the lung can be indirect, and blocking this pathway would be an alternative in order to prevent bronchial vascular remodeling with beneficial clinical results for asthmatic patients (Panariti et al., 2018).

The vascular inflammatory process plays an important role in the process of angiogenesis and vascular remodeling (Feltis et al., 2007; Chan et al., 2019). However, in asthma, the process of angiogenesis is considered complex since it involves many molecules, including cytokines and growth factors, whose individual roles have not yet been fully elucidated. The most potent direct action regulator is known to be VEGF, whose overexpression is often associated with chronic inflammation, fibrosis, and cancer (Chetta et al., 2005). Studies have shown an increase in VEGF in the airway of asthmatic patients compared to healthy controls, with a close positive correlation between the number of VEGFs and an increase in vascularity (Goldie et al., 1995; Liekens et al., 2001; Possa et al., 2012). Feltis et al. (2007) observed an increase in the number of new vessels in asthmatic patients (Feltis et al., 2007). This increase in shoots per vessel was positively correlated to VEGF expression, providing evidence of its roles as an important factor during angiogenic processes. Our data demonstrated an increase in VEGF expression both in the allergic inflammation group and in the LPS exacerbated model. However, we did not observe an increase in new vessels in the OVA group, only in the OVA–LPS group, and treatment with anti-IL-17 was not able to reverse this process. The angiogenesis in the OVA–LPS group can be justified by the use of LPS. Ma et al. (Sakoda et al., 2016) demonstrated that LPS induces new vessel growth, corroborating these findings (Sakoda et al., 2016).

Regarding the role of IL-17 on VEGF expression, Lu et al. (2015) demonstrated that IL-17 treatment in cell cultures leads to an increased VEGF expression, which was attenuated by anti-IL-17 (Lu et al., 2015). These data are in accordance with our findings, demonstrating that anti-IL-17 is able to control VEGF expression in the vascular response.

We hypothesized that the increase of VEGF in the perivascular region was due to TGF-*β* since it is the main factor inducing the expression of VEGF in endothelial cells and fibroblasts (Puxeddu et al., 2005). We also observed an increase in both TNF-a, IL-6, and iNOS, which also contribute to an increase in VEGF expression and aggravate the vascular inflammatory response.

### Effect of Anti-IL-17 Treatment on Immune Cells

In the aspect of inflammatory markers in the different profiles Th1, Th2 and Th17, APC, and regulatory T cells, we observed an increase in the OVA group, and some markers were enhanced in the OVA–LPS group. However, anti-IL-17 was effective in attenuating and controlling different aspects, except for CD8.

As a useful and safe experimental model for studying host responses that suffer from a bacterial infection, we decided to use the same protocol previously used by our group to verify its contribution to the exacerbation of vascular responses (Camargo et al., 2018).

Researchers have become interested in investigating the role of the Th17 profile in chronic allergic inflammation as severely asthmatic patients with different phenotypes have been found to experience patterns of inflammation that cannot be justified by the classic Th2 profile (De Boever et al., 2014). Studies have shown that severe asthmatic patients have an increased neutrophil response, an increase in IL-17 levels, an increased risk of exacerbations, and a low response to the use of corticosteroids. Thus, IL-17 can be a therapeutic target for the treatment of pulmonary inflammation in these patients (Chakir et al., 2003; Hayashi et al., 2013; Pinheiro et al., 2015).

We demonstrated an increase in the number of neutrophils in the bronchoalveolar lavage, and anti-IL-17 was able to attenuate this marker in the treatment group, in addition to controlling and/or attenuating the number of positive cells involved in the various asthmatic response pathways.

Dos Santos et al. (2018) used anti-IL-17 alone and/or associated with the Rho kinase inhibitor to demonstrate that treatment with anti-IL-17 alone or in combination contributed to the control of the disease, demonstrating its efficacy and safety in this experimental model (Dos Santos et al., 2018).

Our data show an important role for the participation of Tregs in the vascular inflammation process observed by the FoxP3+, TGF-*β*, IL-10, and CD4+ data. We did not perform immunohistochemistry for CD25+, a limitation of the study, but the IL-2 data indirectly demonstrate the participation of this pathway in the process of allergic inflammation. Interleukin 2 (IL-2) appears to play an important role in the development of Tregs. Experiments with mice demonstrated that deficiency in both the cytokine IL-2 and the receptor resulted in severe Tregs defects, which was also observed in patients with congenital CD25 molecule deficiency (Murugaiyan et al., 2009; Peters and Yosef, 2014). It is worth mentioning that anti-IL-17 was able to control the inflammatory process, and we hypothesized that the decrease in these Treg markers, especially FoxP3 in the treated groups, was secondary to the decrease in the inflammatory process.

Although we observed an increase in dendritic cells in the OVA and OVA–LPS groups, we emphasize that during the immunohistochemistry process we use an antibody (DC-STAMP) which does not stain B cells, which is also important in the signaling process.

We observed that anti-IL-17 managed to control and attenuate different cells of the vascular inflammatory process in different profiles, Th1, Th2, and Th17. These data corroborate with the control and attenuation found in other studies that evaluate beyond vascular responses (Righetti et al., 2014; Lu et al., 2015; Camargo et al., 2018; Dos Santos et al., 2018). Chan et al. (Chan et al., 2019) demonstrated the importance of the IL-33-ST2 axis in changes in remodeling, angiogenic factors, and angiogenesis (Chan et al., 2019). We demonstrated that anti-IL-17 was able to control this pathway and hypothesized that the attenuation of inflammatory cells in the Th2 profile may have been due to the control of the IL-33-ST2 and TSLP axis; however, more research is needed to confirm these findings.

### Effect of Anti-IL-17 Treatment on Signaling Pathways and Oxidative Stress

We have previously demonstrated the contribution of signaling pathways to asthmatic responses (Possa et al., 2012; Camargo et al., 2018; Dos Santos et al., 2018). The ROCK1 and ROCK2 effectors of the Rho kinase pathway play the role of molecular switches in response to the cell surface receptors of various cytokines, growth factors, and adhesion molecules. However, the relationship between RhoA control in the multiple stages involved in blood vessel formation and its contribution to pulmonary vascular changes is not yet known. Hoang et al. (2004) and Connolly and Aaronson (2011) demonstrated that Rho signaling is supposedly essential for VEGF-dependent angiogenesis in both *in vivo* and *in vitro* (Hoang et al., 2004; Connolly and Aaronson, 2011). In our results, we observed an increase in the expression of ROCK1- and ROCK2-positive cells in the pulmonary vessels, as well as an attenuation of these responses after treatment with anti-IL-17. Bryan et al. (2010) used a pharmacological inhibitor Y-27632 and demonstrated that the inhibition of the Rho Kinase pathway contributed to the control of angiogenesis and VEGF expression (Bryan et al., 2010). Dos Santos et al. (2018) also demonstrated that, when combined with Y-27632 and anti-IL-17, it was able to attenuate ROCK1 and ROCK2 expression in both the airway and lung parenchyma (Dos Santos et al., 2018).

In addition to the Rho kinase pathway, the NF-kb pathway participates in the process of vascular pulmonary inflammation as observed by Sakoda et al. (2016). In their study, they observed an increased NF-kB and VEGF in the pulmonary vessels in the allergic inflammation model, which correlates with the current findings in the present study.

The Jak-Stat signaling pathway, in particular STAT-3, plays an important role in vascular remodeling and angiogenesis *via* stimulation of VEGF, IL-6, IL-8, and IL-17 (Huang et al., 2016). Recently, several studies on the angiogenic process in pulmonary adenocarcinoma biopsies have revealed the participation of STAT1 in this process, wherein IL-17 was found to directly activate tyrosine phosphorylation in the Jak-Stat pathway, possibly stimulating the STAT-1 pathway (Murugaiyan et al., 2009; Peters and Yosef, 2014; Huang et al., 2016). Since the activation of IL-17 *via* STAT-1 is not well documented with regard to allergic inflammatory disease, we evaluated the stimulation of this pathway in our model. As a result, we found an increase in STAT-1 in the OVA group and a potentiation in the OVA–LPS group. However, in terms of STAT phosphorylation, there was little difference between the OVA and OVA–LPS groups. Nevertheless, anti-IL-17 was able to control this signaling pathway.

Nitric oxide (NO) plays an important role in the process of allergic inflammation and remodeling (Prado et al., 2011). It is known that bronchial blood flow correlates positively with exhaled nitric oxide and breathing temperature in individuals with asthma, and these, therefore, can be markers of inflammation and tissue remodeling (Aldakheel et al., 2016). We decided to evaluate NOex, iNOS, and 8-iso-PGF2*α* oxidative stress markers; we observed an increase in the sensitized groups, and the anti-IL-17 was able to attenuate these markers in the treatment groups.

This study has some limitations. One is that there are differences between the model of allergic inflammation in mice compared to human asthma. However, the animal model has become useful, reliable and relevant because clinical studies of asthma are not able to clarify all aspects of the pathophysiology of the disease. In order to ensure the effect of anti-IL-17 treatment on future therapies, models evaluating the effect of exacerbation after 24 h are necessary. Although initially only immunohistochemistry was used for the analysis, another evaluation method could be used to strengthen our findings. However, we recently published that comparative analyses of PCR, ELISA, and immunohistochemistry in this model were similar. The process of chronic allergic inflammation is complex and becoming challenging due to the variety of pathways and processes that are involved. However, these data come to indicate the importance of investigating pulmonary vascular changes in this disease since the process of vascular inflammation in interaction with signaling pathways and oxidative stress results in pulmonary vascular remodeling, contributing to asthmatic responses. In addition, our results provide a potential basis for exploring new therapeutic approaches to the treatment of asthma.

## Conclusions

In this model of LPS-exacerbated chronic allergic inflammation, IL-17 inhibition represents a promising therapeutic strategy since it contributes to the bronchial vascular control of the different inflammatory profiles Th1, Th2, and Th17, pulmonary mechanics, as well as the remodeling and activation of the oxidative stress pathway. Our findings suggest that this effect may be associated with the control of different signaling pathways.

## Data Availability Statement

The raw data supporting the conclusions of this article will be made available by the authors, without undue reservation, to any qualified researcher.

## Ethics Statement

The animal study was reviewed and approved by the Research Ethics Committee of the Hospital das Clínicas of the Medical School of the University of São Paulo (protocol number 141/16). This work was developed in the Laboratory of Experimental Therapy I (LIM 20) of the Faculty of Medicine of the University of São Paulo.

## Author Contributions

IT and LC conceived the study. RR, TS, and SF performed the experiments. LC, RR, TS, FA, and SF analyzed the data. IT, RR, FL, CP, EL, and MM contributed reagents/materials/analysis tools. IT, RR, and LC wrote the paper. All authors contributed to the article and approved the submitted version.

## Funding

The authors thank the Fundação de Amparo à Pesquisa do Estado de São Paulo (FAPESP), grant 2018/02537-5, the Conselho Nacional de Desenvolvimento Científico e Tecnológico (CNPq), the Coordenação de Aperfeiçoamento de Pessoal de Nível Superior (CAPES), and the Laboratory of Medical Investigations (LIM-20 FMUSP) for their financial support.

## Conflict of Interest

The authors declare that the research was conducted in the absence of any commercial or financial relationships that could be construed as a potential conflict of interest.
